# Chemokine Profiles Are Affected in Serum of Patients with Acute Rejection of Kidney Allograft

**DOI:** 10.1155/2021/5513690

**Published:** 2021-03-11

**Authors:** Lenka Krupickova, Martina Fialova, Marek Novotny, Veronika Svachova, Kristyna Mezerova, Eva Cecrdlova, Ondrej Viklicky, Ilja Striz

**Affiliations:** ^1^Department of Clinical and Transplant Immunology, Institute for Clinical and Experimental Medicine, Videnska 1958/9 Prague, Czech Republic; ^2^Transplant Center, Department of Nephrology, Institute for Clinical and Experimental Medicine, Videnska 1958/9 Prague, Czech Republic

## Abstract

Kidney allograft transplantation improved the prognosis and quality of life of patients with end-stage renal diseases but the occurrence of acute rejection represents a limitation of the final outcome. Noninvasive biomarkers are needed as well as further advancements in the understanding of immune mechanisms of reaction to the allograft. Our study of 138 patients focused on one-year monitoring of serum concentrations of 12 chemokines regulating the recruitment of different immune cells into transplanted allograft and on *in vitro* regulation of the same chemokines release by interactions of renal proximal epithelial cells with monocyte/macrophage cell line stimulated with TNF alpha. In a group of 44 patients with acute rejection, higher serum pretransplant levels of CXCL1, CXCL5, CXCL6, CCL2, CCL21, and particularly CXCL10 and CX3CL1(both *p* < 0.001) were found suggesting their higher proinflammatory status as compared to subjects with the uncomplicated outcome. In samples collected at the day of biopsy positive for acute rejection, chemokines CXCL9 and CXCL11 attracting preferentially Th1 lymphocytes were found to be upregulated. In our *in vitro* model with TNF alpha induction, renal proximal epithelial cells seemed to be a more potent source of chemokines attracting neutrophils as compared to monocyte/macrophage cell line but the coculture of these cells potentiated release of neutrophilic chemokines CXCL5 and CXCL6. Similar augmentation of chemokine production was found also in the case of CCL2. On the other hand, adding of monocytes/macrophages to a culture of renal epithelial cells suppressed the release of CXCL10 and CXCL11 attracting T lymphocytes. We assume from our data that in kidney allograft transplantation, chemokines attracting neutrophils, T lymphocytes, and monocytes are induced simultaneously and measurement some of them in combination might be used as biomarkers of acute rejection. Mutual cell-cell interactions of immune cells with renal parenchyma seem to be important for fine regulation of chemokine release.

## 1. Introduction

Kidney allograft transplantation is widely accepted to be the treatment of choice in patients with end-stage renal diseases by improving the quality of life and prolongation of survival compared to dialysis. Advancements in the development of immunosuppressive agents for induction and maintenance therapy are associated with better graft survival but alloreactive immune mechanisms may lead to acute rejection in a nonnegligible number of patients. The diagnosis of acute rejection is usually suspected in a relatively advanced stage based on kidney function parameters (elevated serum urea and creatinine, proteinuria) and requires confirmation by a kidney biopsy. This invasive procedure does not have the optimal reproducibility due to focal characteristics of immune responses and subjective evaluation of samples by pathologists regardless of intensive efforts to constantly improve international guidelines established by Banff classification [1]. In this respect, evaluation of gene-expression signatures in biopsy samples represents one of the promising approaches [2] but there is an obvious need for noninvasive biomarkers of rejection [3].

Kidney transplantation is associated with an immediate influx of immune cells into the allograft mediated by upregulation of endothelial adhesion molecules and release of chemokines from parenchymal cells. Chemokines are a family of cytokines with low molecular weight which enables them optimal penetration required to form tissue gradient to recruit inflammatory cells to the site of injury or immune response [4]. They are traditionally classified into four families (CXC, CC, CX3X, and XC) based on the arrangements of the two N-terminal cysteine residues [5, 6] and bind to G protein-coupled receptors expressed on a wide variety of cells [7]. According to their function, chemokines are generally referred to as being either inflammatory or homeostatic [8]. Under inflammatory conditions, neutrophil recruitment is regulated mainly by mediators secreted from epithelial cells and chemokines are supposed to play a key role [9] [10]. It has been shown that unstimulated epithelial cells constitutively express mRNA of chemokines attracting preferentially neutrophils while chemokines specific for mononuclear phagocytes and lymphocytes are induced after their stimulation with proinflammatory cytokines [11]. Experimental models of kidney allograft transplantation suggest that early chemokine induction occurs in two phases. In the first phase, chemokines attracting neutrophils such as IL-8/CXCL8, MIP-2/CXCL2, and Gro-alpha/CXCL1 predominate, and the second phase is characterized by a release of RANTES/CCL5, MCP-1/CCL2, MIP-1/CCL3, MIG/CXCL9, IP-10/CXCL10, and ITAC/CXCL11 and provide signals for recruitment of monocytes, natural killer (NK) cells, and T lymphocytes [12]. It has been shown in another mouse kidney transplant model that moderate to severe ischemia/reperfusion injury leads to a long-term chemokine upregulation and T cell infiltration of the allograft [13]. In addition to their major role in leukocyte trafficking and homing, chemokines are involved also in other homeostatic and pathophysiological processes [14, 15] and might play an important role also in later phases of the immune response to kidney allograft. Since some of the chemokines provide also profibrotic activity [16], this mechanism might be potentially involved in the pathophysiology of chronic rejection. Our prospective study is aimed at monitoring serum levels of multiple inflammatory chemokines in kidney allograft recipients with respect to the clinical outcome of transplantation and the presence of acute rejection. Furthermore, regulation of chemokine release was studied in a tissue coculture model of human proximal renal epithelial cells with macrophages to find out a prevailing potential source of individual chemokines and a role of mutual cell-cell interactions in their production.

## 2. Patients and Methods

### 2.1. Patients

A total of 138 patients who underwent renal transplantation from deceased donors at our transplant centre were enrolled as part of the study. The demographic characteristics of the study subjects are shown in Table 1. All patients provided their informed consent after the study protocol was approved by the Ethics Committee of the Institute for Clinical and Experimental Medicine (ID 118099).

### 2.2. Induction Treatment and Maintenance Immunosuppression

All patients received induction immunosuppressive therapy according to the centre's immune risk-based protocol. Primary kidney transplant recipients with PRA < 20% and negative DSA received basiliximab (*n* = 43). Patients with PRA > 20% received rabbit antithymocyte globulin (*n* = 77). Individuals confirmed as DSA-positive at the time of transplantation (*n* = 18) underwent plasma exchange (PE) prior to transplantation in addition to intravenous immunoglobulin (IVIG) and ATG. The majority of patients (*n* = 125) were treated with triple maintenance therapy consisting of tacrolimus, mycophenolate mofetil and corticosteroids. Ten patients were placed on dual therapy with tacrolimus and corticosteroids. Three patients were converted to everolimus in combination with mycophenolate mofetil and corticosteroids early after transplantation.

### 2.3. Histopathology and Rejection Phenotypes

Kidney allograft biopsy samples were obtained using a percutaneous ultrasound-guided 16G biopsy needle for for-cause or protocol biopsy, procedures routinely performed 3 months posttransplant in our centre. All patients provided their informed consent and signed an agreement upon the performance of each biopsy. Biopsy-proven acute rejection was diagnosed histologically according to the revised Banff 2017 classification [1]. Out of 44 patients with acute rejection, 16 had T cell-mediated rejection characterized by tubulointerstitial inflammation or intimal arteritis in the absence of C4d and DSA. Twenty-eight patients had histological signs of antibody-mediated rejection such as glomerulitis, peritubular capillaritis, and/or intimal arteritis in the presence of C4d. The microvascular inflammation score was at least 2 in the absence of C4d. While DSAs were detectable in 13 patients, they went undetected in the remaining 15 patients. Diagnosis of antibody-mediated rejection was based on histology.

### 2.4. THP-1 Cocultures with Renal Proximal Tubular Epithelial Cells

Renal proximal tubular epithelial cells (RPTEC, from ATCC) were cultured in Iscove's modified Dulbecco's medium (IMDM) with 10% fetal calf serum (FCS) and L-glutamine, penicillin, and streptomycin (Sigma-Aldrich, MO, USA) until confluency in 6-well tissue culture plates. The medium was removed, and the cells were washed twice with Earle's Balanced Salt Solution (EBS) to exclude the effect of different serum factors before the cocultures. THP-1 monocyte/macrophage cell line (from ATCC) was cultured in the same medium as RPTEC and additionally supplemented with mercaptoethanol. The cells were physically separated with 0.4 *μ*m pore filters to avoid tight contact with each other (Corning, NY, USA). Cocultured cells were stimulated with proinflammatory cytokine TNF alpha (R&D Systems (Minneapolis, MN, USA)) at 10 ng/ml for 24 h to induce the release of chemokines into culture media.

### 2.5. Chemokine Analysis

We used xMAP (multianalyte profiling) technology from Luminex Corporation for simultaneous detection of 12 chemokines (CXCL1, CXCL5, CXCL6, CXCL8, CXXCL9, CXCL10, CXXCL11, CXCL16, CCL2, CCL5, CCL21, and CX3CL1) in the sera of kidney transplant recipients and in culture supernatants using the Human magnetic Luminex assay kit from R&D Systems (Minneapolis, MN, USA) and the instrument Luminex 200. Briefly, during the first 2 hours, 50 *μ*l of serum or supernatants is incubated at room temperature with gentle agitation on a horizontal orbital shaker together with chemokine-specific antibody-coated microparticles, and after washing of unbound substances, the secondary antibodies conjugated with biotin are added and samples are then incubated for another 1 hour. After incubation and washing of unbound secondary antibodies, streptavidin-PE is added which binds to biotin on a secondary biotinylated antibody and the microparticles are, after 30 minutes of incubation and washing, finally resuspended in 100 *μ*l of wash buffer and then evaluated by the Luminex analyzer. Standards and samples are incubated in special filter-bottomed 96-well plates from which the fluid is drained out using a microplate vacuum manifold. The concentrations of individual chemokines were calculated by interpolation from particular standard curves.

### 2.6. Statistics

Statistical analyses were performed by the GraphPad Prism 5 software (GraphPad Software, La Jolla, CA, USA). Based on the distribution of the data, parametric or nonparametric (Mann-Whitney and Kruskal-Wallis) testing was used. We performed logarithmical transformation and parametric testing by repeated measure ANOVA when comparing the data in different time points.

## 3. Results

### 3.1. Serum Levels of Chemokines Attracting Mainly Neutrophils (Gro-Alpha/CXCL1, ENA-78/CXCL5, GCP-2/CXCL6, and IL8/CXCL8) in Kidney Transplant Recipients

**Figure 1:**
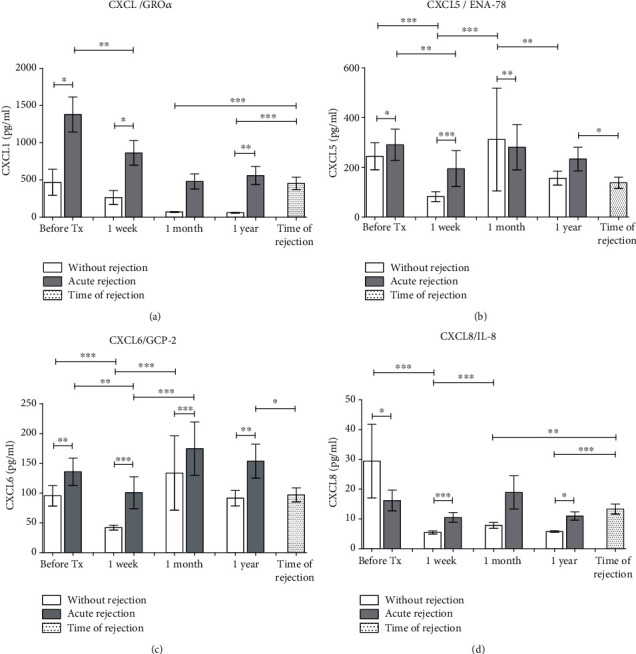
Serum concentrations of chemokines attracting mainly neutrophils (a) CXCL1, (b) CXCL5, (c) CXCL6, and (d) CXCL8 were measured in samples collected before and one week, one month, and one year after kidney allograft transplantation. Results from patients with uncomplicated outcomes (*n* = 94) were compared with those with acute rejection (*n* = 44). In patients with acute rejection, additional serum sample was collected at the day of diagnostic biopsy positive for rejection. Data are expressed as mean ± SEM; ^∗^*p* < 0.05; ^∗∗^*p* < 0.01; ^∗∗∗^*p* < 0.001.

The serum concentrations of chemokines with ability to bind either to CXCR2 (chemokine CXCL1) or both CXCR1 and CXCR2 (chemokines CXCL5, CXCL6, and CXCL8) expressed on neutrophils were measured in patients before kidney allograft transplantation and then at one week, one month, and one year. In patients with acute rejection, additional serum sample was collected at the day of diagnostic biopsy positive for rejection. We have found higher pretransplant concentrations of CXCL1 in patients with acute rejection, and increased levels of this chemokine in comparison with patients with uncomplicated outcome were detected at one week, one month, one year, and also in the time of rejection, although the concentration of CXCL1 decreased during the first month after the transplantation and further at one month (Figure 1(a)). Concentrations of CXCL5 decreased during the first week after the transplantation of both groups, and patients with acute rejection had higher pretransplant values (Figure 1(b)). Higher levels of CXCL5 were detected in patients with acute rejection than in those with the uncomplicated outcome at one week and one month with an inverse relationship at one month. In samples collected at the time of rejection, serum concentrations of CXCL5 were not upregulated. Similar profile with posttransplant downregulation of serum levels was found also in the case of CXCL6 (Figure 1(c)). In patients with acute rejection, a higher concentration of CXCL6 was found at all time points as compared to subjects with the uncomplicated outcome. Upregulation of CXCL8 in patients with acute rejection in comparison to subjects with normal outcome was found at one week and one month after the transplantation and the time of biopsy while the pretransplant values were higher in patients with the uncomplicated outcome (Figure 1(d)).

### 3.2. Chemokines Attracting Mainly T Lymphocytes (MIG/CXCL9, IP-10/CXCL10, ITAC/CXCL11, CXCL16, and 6Ckine/CCL21)

**Figure 2:**
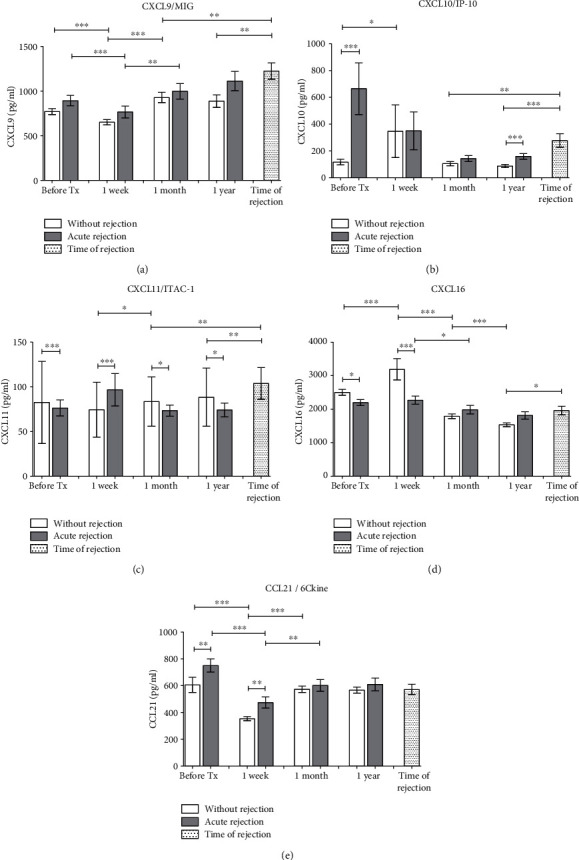
Chemokines recruiting preferentially T lymphocytes (a) CXCL9, (b) CXCL10, (c) CXCL11, and (d) CXCL16 were measured in sera obtained from patients with normal outcomes (*n* = 94) and those with acute rejection (*n* = 44) before and one week, one month, and one year after kidney transplantation surgery. In patients with acute rejection, additional data were obtained from blood on the day a positive diagnostic kidney biopsy was confirmed. Data are expressed as mean ± SEM; ^∗^*p* < 0.05; ^∗∗^*p* < 0.01; ^∗∗∗^*p* < 0.001.

Serum samples collected before the kidney transplantation and then after one week, one month, one year, and at the time of biopsy positive for rejection were tested for concentration of chemokines with the ability to bind receptors CXCR3 (chemokines CXCL9-11), CXCR16 (chemokine CXCL16), and CCR7 (chemokine CCL21). Concentrations of CXCL9 were transiently decreased at one week after the transplantation with consequent recovery to pretransplant levels at one month (Figure 2(a)). In samples taken at the day of diagnostic biopsy positive for rejection, serum levels of CXCL9 were higher than in all posttransplant time points of subjects with the uncomplicated outcome. Pretransplant concentrations of CXCL10 (Figure 2(b)) were much higher in subjects with acute rejection than in those with the uncomplicated outcome but there was no difference observed at one week and one month after the transplantation between those two groups of patients. CXCL10 concentrations in samples taken at the time of biopsy diagnostic for rejection were higher than those of patients with the uncomplicated outcome at one month and one year. The serum concentration of CXCL11 was upregulated in patients with acute rejection at one week after the transplantation and at the time of biopsy positive for rejection, and in other time points, CXCL11 concentrations were higher in patients with the uncomplicated outcome (Figure 2(c)). Serum concentrations of CXCL16 increased during the first week after the kidney allograft transplantation only in patients with the uncomplicated outcome but decreased markedly during the first month and with no change in none of two groups after one year. In samples taken at the time of rejection, the concentration of CXCL16 was higher than in subjects with the uncomplicated outcome at one year (Figure 2(d)). Concentrations of CCL21 (Figure 2(e)) decreased during the first week after kidney allograft transplantation in both groups, and the pretransplant levels were higher in patients with acute rejection.

### 3.3. Chemokines Attracting Mainly Monocytes, NK Cells, Eosinophils, and Basophils (MCP-1/CCL2, Fractalkine/CX3CL1, and RANTES/CCL5)

**Figure 3:**
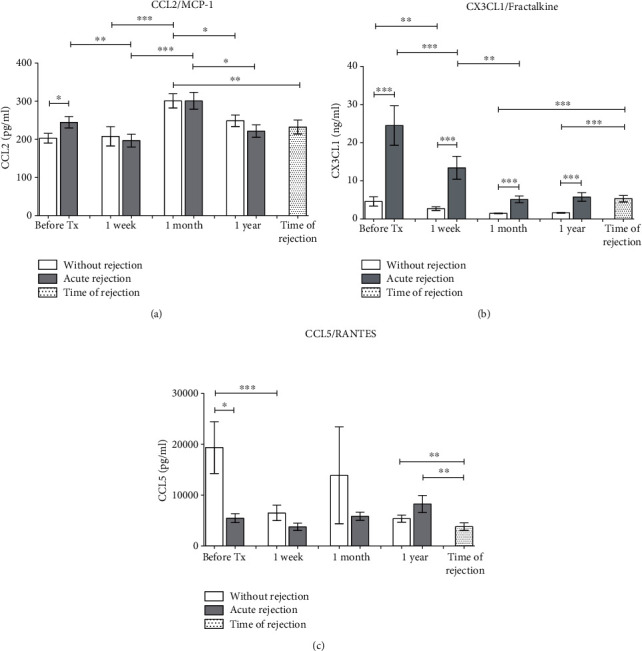
Concentrations of chemokines attracting mainly monocytes, NK cells, eosinophils, and basophils (a) CCL2, (b) CX3CL1, and (c) CCL5 were evaluated in sera of kidney transplant patients with uncomplicated outcomes (*n* = 94) and those with acute rejection (*n* = 44) before and one week, one month, and one year after surgery. In addition, blood was drawn also at the day of diagnostic biopsy positive for acute rejection. Data are expressed as mean ± SEM; ^∗^*p* < 0.05; ^∗∗^*p* < 0.01; ^∗∗∗^*p* < 0.001.

To compare results of chemokine concentrations in uncomplicated patients after kidney allograft transplantation with those with acute rejection, serum was obtained before the surgery and then after one week, one month, and one year. In addition, blood was drawn also at the day of diagnostic biopsy positive for acute rejection. The concentration of CCL2, a chemokine with ability to bind receptors CCR2 and CCR4, increased transiently at one month after the transplantation without clear differences between both groups of patients (Figure 3(a)). The CX3CL1 chemokine related to a receptor CX3CR showed increased pretransplant concentrations in patients with acute rejection as compared to subjects with the uncomplicated outcome of kidney allograft transplantation (Figure 3(b)). At all time points including the time of diagnostic biopsy positive for acute rejection, CX3CL1 levels were higher in the rejection group than in patients with the normal outcome. In contrast, CCL5 chemokine with ability to bind chemokine receptors CCR3, CCR5, and CCR1 was higher in patients with the uncomplicated outcome than in rejection group before the kidney transplantation and then one week and one month after the surgery (Figure 3(c)). CCL5 levels in sera collected at the time of biopsy positive for rejection were lower than those subjects with the uncomplicated outcome at all time points.

### 3.4. *In Vitro* Production of Chemokines Attracting Mainly Neutrophils (Gro-Alpha/CXCL1, ENA-78/CXCL5, GCP-2/CXCL6, and IL8/CXCL8)

Renal proximal epithelial cells (RPTECs) were cultured until confluency and then stimulated with TNF alpha (10 ng/ml) in the absence or presence of monocyte/macrophage cell line THP-1 separated by a filter insert. Separately, a culture of THP-1 cells was stimulated with TNF alpha, too. After 24 h, culture supernatants were tested for chemokines concentrations.

**Figure 4:**
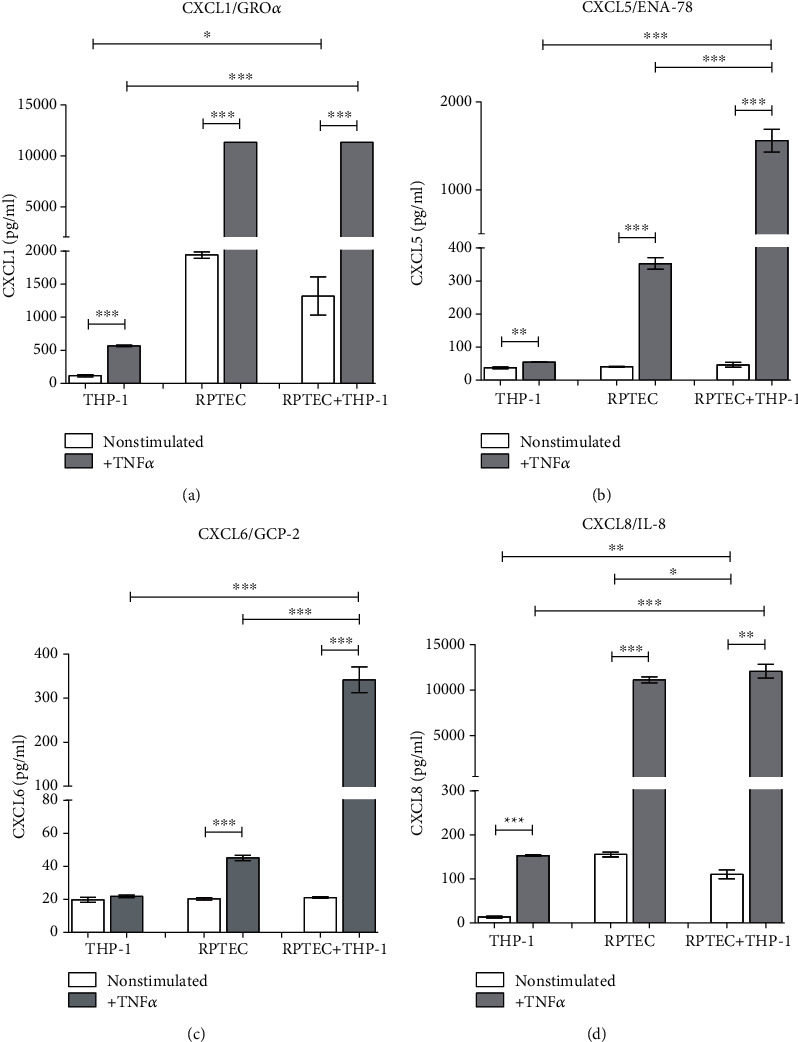
Renal proximal tubular cells (RPTECs) were cultured until their confluency and then cocultured with THP-1 cells (monocyte/macrophage cell line separated with 0.4 *μ*m pore filters, stimulated with TNF alpha (10 ng/ml)). After 24 hours, the supernatants were collected and concentrations of chemokines attracting mainly neutrophils (a) CXCL1, (b) CXCL5, (c) CXCL6, and (d) CXCL8 were measured. Data represent one of repetitive experiments done in triplicate and are expressed as mean ± SEM; ^∗^*p* < 0.05; ^∗∗^*p* < 0.01; ^∗∗∗^*p* < 0.001.

RPTECs produced much higher concentrations of CXCL1 as compared to THP-1 cells, and a coculture with monocytes did not affect the release of this cytokine induced by TNF alpha (Figure 4(a)). Also, in CXCL5, RPTCs represented the main source of the chemokine, while THP-1 cells did not respond to TNF alpha stimulation. On the other hand, coculture with THP-1 increased the TNF alpha-induced release of CXCL5 from RPTECs dramatically (Figure 4(b)). Although the basal production of CXCL6 did not differ between the cell types, only RPTECs responded to the TNF alpha stimulation and a coculture with THP-1 further upregulated CXCL6 release (Figure 4(c)). Both THP-1 cells and RPTECs responded to TNF alpha stimulation by the release of CXCL8, and its maximal concentration produced by RPTECs was not affected by the coculture (Figure 4(d)).

### 3.5. *In Vitro* Production of Chemokines Attracting Mainly T Lymphocytes (MIG/CXCL9, IP-10/CXCL10, ITAC/CXCL11, CXCL16, and 6Ckine/CCL21)

**Figure 5:**
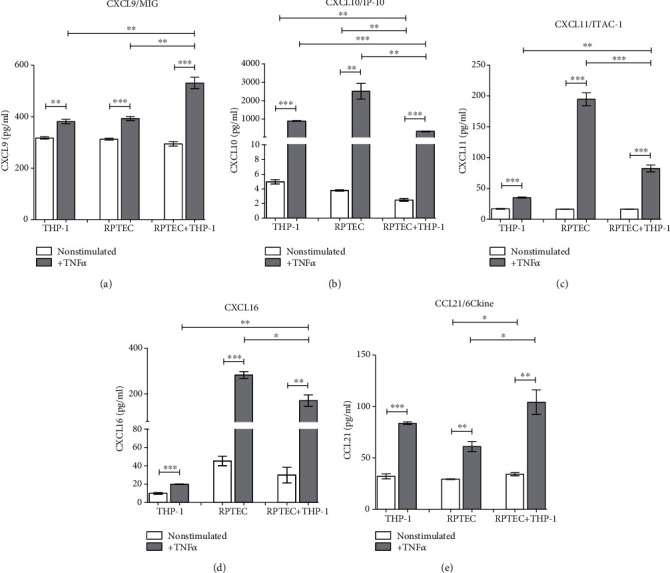
In vitro production of chemokines attracting mainly T lymphocytes (a) CXCL9, (b) CXCL10, (c) CXCL11, (d) CXCL16, and (e) CCL21 were measured in TNF alpha- (10 ng/ml) induced isolated cultures of confluent RPTECs and THP-1 cells or in their 24 h cocultures. Data represent one of repetitive experiments done in triplicate and are expressed as mean ± SEM; ^∗^*p* < 0.05; ^∗∗^*p* < 0.01; ^∗∗∗^*p* < 0.001.

CXCL9 release was induced by TNF alpha in both cell types with an additive effect of a coculture (Figure 5(a)). Chemokines CXCL10, CXCL11, and CXCL16 (Figures 5(b)–5(d)) were induced by TNF alpha in both cell types but coculture with THP-1 cells downregulated their release as compared to the highest induction in RPTC alone culture. In contrast, CCL21 release was upregulated in by TNF alpha in both cell types and with an additive effect of a coculture (Figure 5(e)).

### 3.6. *In Vitro* Production of Chemokines Attracting Mainly Monocytes, NK Cells, Eosinophils, and Basophils (MCP-1/CCL2, Fractalkine/CX3CL1, and RANTES/CCL5)

**Figure 6:**
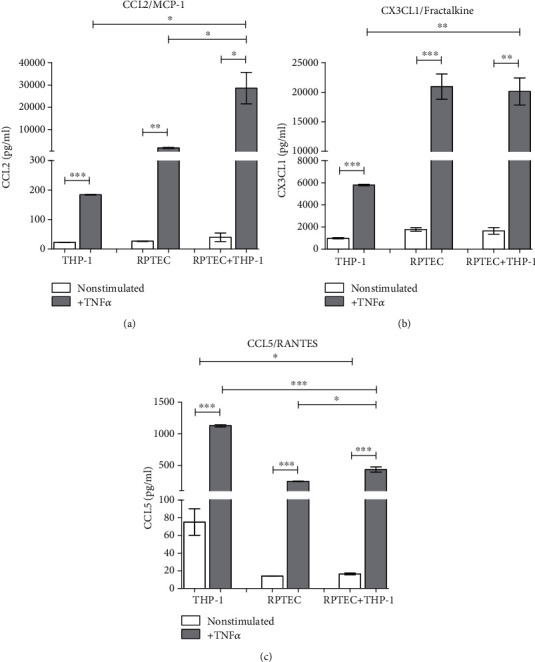
Concentrations of chemokines attracting mainly monocytes, NK cells, eosinophils, and basophils (a) CCL2, (b) CX3CL1, and (c) CCL5 were measured in cultures of confluent RPTECs, THP-1 cell line, or their 24 h cocultures under the presence or absence of TNF alpha (10 ng/ml). Data represent one of repetitive experiments done in triplicate and are expressed as mean ± SEM; ^∗^*p* < 0.05; ^∗∗^*p* < 0.01; ^∗∗∗^*p* < 0.001.

CCL2 chemokine was induced by TNF alpha only in RPTECs but coculture with THP-1 cells augmented its release (Figure 6(a)). Also, the induction of CX3CL1 by TNF alpha was demonstrated only in renal epithelial cells and not in THP-1 macrophages, and no upregulation was observed by a coculture (Figure 6(b)). CCL5 production was increased in response to TNF alpha in both cell types but in a coculture with renal epithelial cells, THP-1 macrophages secreted less chemokine than in a simple culture (Figure 6(c)).

## 4. Discussion

Kidney allograft transplantation is followed by consequent massive recruitment of different immune cells into a transplanted organ, and our study demonstrated the association of the development of acute rejection with increased serum concentrations of several chemokines attracting neutrophils, lymphocytes, monocytes, and other immunocompetent cells. Dynamic changes in blood concentrations of several chemokines might be used as potential biomarkers of the immune response against kidney allograft. *In vitro* cocultures of renal epithelial cells with monocytes/macrophages suggest that while the release of some chemokines attracting neutrophils may profit from mutual potentiation of these cells, the production of lymphocyte attracting chemokines CXCL10 and CXCL11 from renal epithelial cells is inhibited by the presence of monocytes/macrophages.

From chemokines regulating the recruitment of neutrophils, CXCL1 (Gro-alpha) seems to be the most relevant to mechanisms of acute rejection being found upregulated in comparison to subjects with the normal outcome at all time points including pretransplant concentrations. Obviously, the specificity of this potential biomarker, as well as other chemokines attracting neutrophils, may be affected by the fact that these cells are the first-line defence in antibacterial immunity. In this respect, increased transcripts of CXCL1 gene have been documented also in renal biopsies of patients with acute pyelonephritis [17]. Our *in vitro* experiments demonstrated that coculture of renal proximal tubular epithelial cells with monocyte/macrophage cell line augmented the release of CXCL5 and CXCL6 induced by TNF alpha. Renal epithelial cells seem to be a major source of these two chemokines together with CXCL1 and CXCL8, although these are induced also in monocytes/macrophages. We are aware that using monocyte/macrophage cell line THP-1 may lead to slightly different results than exploring of peripheral blood monocytes but the benefit of an unlimited number of cells of high purity without any need of separation methods prevailed. There are numerous data showing that THP-1 cells in culture experiments resemble cultured peripheral blood monocytes [18].

Our study then demonstrated an association of increased serum levels of chemokines CXCL9, CXCL10, and CXCL11 attracting preferentially T lymphocytes with the acute rejection of kidney allograft being higher in samples collected at the time of confirmed acute rejection as compared to samples from patients with the uncomplicated outcome. Pretransplant elevation of CXCL10 concentration in patients with acute rejection is in agreement with already published data showing its association with the risk of graft failure [19] while the early elevation of CCL21 has not been described, so far. Early higher CXCL10 serum levels are known to be connected with rejection in HLA-incompatible renal transplantation [20]. Urinary CXCL10 levels have been already found to be increased in both acute rejection patients [21] and tubulointerstitial BKV infection [22] or in pediatric kidney allograft transplantation eGFR decline [23]. The urinary CXCL9 concentration increases before the elevation of serum creatinine in patients at risk for acute kidney allograft rejection [24]. The role of CXCL9-11 chemokines in acute rejection of the allograft is supported also by the upregulation of their mRNA in kidney biopsies together with higher expression of their receptor CXCR3 [25]. Expression of the chemokine receptor CXCR3 on plasma cells can be effectively inhibited by an immunoproteasome inhibitor in a rat model of kidney transplantation [26]. Also, blocking of CXCL9 (MIG), a key chemokine regulating the influx of allogeneic T cells into the graft, prolongs allograft survival in an experimental model of heart transplantation [27]. Our study did not confirm previously published data where kidney transplant patients with acute rejection treated with antithymocyte globulin showed increased pretransplant serum levels of CXCL9 [28]. In this respect, no conclusive effect of induction protocols on serum levels of any tested chemokine was found (data not shown). While CXCL9 and CXCL16 are induced by TNF alpha in both epithelial cells and monocyte/macrophages, in the case of CXCL10 and CXCL11, renal epithelium seems to be the main source and monocytes/macrophages downregulate the release of these chemokines. Factors released from activated monocytes/macrophages to downregulate T cell-related chemokines may be multiple, and both proinflammatory and anti-inflammatory cytokines are produced in response to proinflammatory stimuli.

The influx of T lymphocytes together with monocytes can be mediated also by CX3CL1 [29]. Our data suggested the potential role of CX3CL1 (fractalkine) in acute rejection of kidney allograft with increased concentrations of this chemokine at all time points as compared to subjects with the uncomplicated outcome. Very high concentrations of prepretransplant CXCR3L1 in patients with acute rejection are in agreement with other study showing elevation of this cytokine at day 0 as compared with rejection-free subjects [30]. These data support the hypothesis that immune mechanisms activated in the recipient predispose him to a worse outcome. CCL2 (MCP-1, monocyte chemotactic factor) is a chemokine attracting mainly mononuclear phagocytes and seems to be involved also in monocyte-endothelial interactions [31] at least partially by upregulated expression of CD11b integrin expression on “classical” monocytes [32]. The intragraft expression of CCL2 (MCP-1) mRNA has been already described in patients with chronic allograft nephropathy [33]. We have found only moderate pretransplant elevation of this chemokine in the serum of patients with acute rejection as compared to subjects with the uncomplicated outcome. In an experimental model of transplantation tolerance induced by myeloid-derived suppressor cells, development of rejection was associated with an increase of CCL2 transcripts in the kidney together with elevated levels of CCL2 in the blood [34]. Also, mRNA for CCL5 (RANTES) together with other chemokines was found upregulated in kidney biopsies of patients with the acute rejection of the allograft [25]. Chemokines CCL2, CCL5 (RANTES), and CX3CL1 are in vitro inducible by TNF alpha in both renal epithelial cells and monocytes/macrophages with coculture potentiation only in the case of CCL2.

Recent transcriptome profiling of kidney allograft biopsy specimens from patients with TCMR identified heightened expression of multiple chemokines responsible for the recruitment of multiple immune populations (CCL2-4, CXCL1, and CXCL9-11) [35]. Our data demonstrated that heterogeneous chemokine profiles can be observed also in the serum of kidney allograft recipients, and their combination may be thus taken into account as potential noninvasive markers of acute rejection. Targeting chemokines represents also an attractive tool to control immunopathological processes, and chemokine receptor inhibitors are thus extensively studied especially in cancer immunotherapy [36]. In experimental transplant models, a CXCR4 antagonist burixafor significantly attenuated the incidence rate of acute rejection after heart transplantation in minipigs [37] and CXCR4 antagonism inhibited the expression of profibrotic genes and attenuated renal fibrosis in rats [38]. Chemokine production in transplanted kidney is not dependent only on the kind of stimuli, where TNF alpha and IL-1 family cytokines [39] are known to be particularly important. Our tissue culture data suggest that mutual interactions of immune cells such as tissue macrophages or monocytes with renal epithelium may represent another factor for fine regulation of chemokine release and leukocyte recruitment.

## Figures and Tables

**Table 1:** The demographic characteristics and clinical features of study groups.

	Normal outcome	Rejection
Number of patients (*n*)	94	44
*Type of acute rejection (*n*, %)*		
Cellular		16 (36.4)
Humoral/DSA+		13 (29.5)
Humoral/DSA-		15 (34.1)
Gender (male/female) (*n*)	61/33	28/16
Recipient age (median, range)	58 (22-80)	54 (23-79)
Donor age (median, range)	56.5 (1-81)	51 (21-75)
Cold ischemia time (hours) (median, range)	14 (0-24)	15 (0-21)
PRA (%) (median, range)	10 (0-98)	10 (0-92)
HLA mismatches (median, range)	3 (1-6)	3 (0-6)
*DSA positivity (*n*, %)*		
Preformed DSA	8 (8.5)	14 (31.8)
*De novo* DSA	0	5 (11.4)
*Induction therapy (*n*, %)*		
Basiliximab	27 (28.7)	16 (36.4)
ATG	59 (62.8)	18 (40.9)
ATG, PE, IVIG	8 (8.5)	10 (22.7)
*Maintenance IS (*n*, %)*		
TAC, MMF, CS	86 (91.5)	39 (88.6)
TAC, KS	5 (5.3)	5 (11.4)
mTOR, MMF, CS	3 (3.2)	0 (0)

## Data Availability

The data used to support the findings of this study are available from the corresponding author upon request.

## References

[1] Haas M., Loupy A., Lefaucheur C. (2018). The Banff 2017 Kidney Meeting Report: revised diagnostic criteria for chronic active T cell-mediated rejection, antibody-mediated rejection, and prospects for integrative endpoints for next-generation clinical trials. *American Journal of Transplantation*.

[2] Madill-Thomsen K., Perkowska-Ptasińska A., Böhmig G. A. (2020). Discrepancy analysis comparing molecular and histology diagnoses in kidney transplant biopsies. *American Journal of Transplantation*.

[3] Eikmans M., Gielis E. M., Ledeganck K. J., Yang J., Abramowicz D., Claas F. F. J. (2019). Non-invasive biomarkers of acute rejection in kidney transplantation: novel targets and strategies. *Frontiers in Medicine*.

[4] Ono S. J., Nakamura T., Miyazaki D., Ohbayashi M., Dawson M., Toda M. (2003). Chemokines: roles in leukocyte development, trafficking, and effector function. *The Journal of Allergy and Clinical Immunology*.

[5] Nomiyama H., Osada N., Yoshie O. (2013). Systematic classification of vertebrate chemokines based on conserved synteny and evolutionary history. *Genes to Cells*.

[6] Blanchet X., Langer M., Weber C., Koenen R. R., von Hundelshausen P. (2012). Touch of chemokines. *Frontiers in Immunology*.

[7] Bachelerie F., Ben-Baruch A., Burkhardt A. M. (2014). International Union of Basic and Clinical Pharmacology. [corrected]. LXXXIX. Update on the extended family of chemokine receptors and introducing a new nomenclature for atypical chemokine receptors. *Pharmacological Reviews*.

[8] Dyer D. P., Medina-Ruiz L., Bartolini R. (2019). Chemokine receptor redundancy and specificity are context dependent. *Immunity*.

[9] Szabady R. L., McCormick B. A. (2013). Control of neutrophil inflammation at mucosal surfaces by secreted epithelial products. *Frontiers in Immunology*.

[10] Sanz M. J., Kubes P. (2012). Neutrophil-active chemokines in in vivo imaging of neutrophil trafficking. *European Journal of Immunology*.

[11] Thorburn Nee Krasna E., Kolesar L., Brabcova E. (2009). CXC and CC chemokines induced in human renal epithelial cells by inflammatory cytokines. *APMIS*.

[12] de Vries V. C., Elgueta R., Lee D. M., Noelle R. J. (2010). Mast cell protease 6 is required for allograft tolerance. *Transplantation Proceedings*.

[13] Ascon M., Ascon D. B., Liu M. (2009). Renal ischemia-reperfusion leads to long term infiltration of activated and effector-memory T lymphocytes. *Kidney International*.

[14] Chen K., Bao Z., Tang P., Gong W., Yoshimura T., Wang J. M. (2018). Chemokines in homeostasis and diseases. *Cellular & Molecular Immunology*.

[15] Ridiandries A., Tan J. T. M., Bursill C. (2018). The Role of Chemokines in Wound Healing. *International Journal of Molecular Sciences*.

[16] Sahin H., Wasmuth H. E. (2013). Chemokines in tissue fibrosis. *Biochimica et Biophysica Acta*.

[17] Oghumu S., Nori U., Bracewell A. (2016). Differential gene expression pattern in biopsies with renal allograft pyelonephritis and allograft rejection. *Clinical Transplantation*.

[18] Bosshart H., Heinzelmann M. (2016). THP-1 cells as a model for human monocytes. *Annals of Translational Medicine*.

[19] Rotondi M., Rosati A., Buonamano A. (2004). High pretransplant serum levels of CXCL10/IP-10 are related to increased risk of renal allograft failure. *American Journal of Transplantation*.

[20] Field M., Lowe D., Cobbold M. (2014). The use of NGAL and IP-10 in the prediction of early acute rejection in highly sensitized patients following HLA-incompatible renal transplantation. *Transplant International*.

[21] Matz M., Beyer J., Wunsch D. (2006). Early post-transplant urinary IP-10 expression after kidney transplantation is predictive of short- and long-term graft function. *Kidney International*.

[22] Ho J., Schaub S., Wiebe C. (2018). Urinary CXCL10 chemokine is associated with alloimmune and virus compartment-specific renal allograft inflammation. *Transplantation*.

[23] Mockler C., Sharma A., Gibson I. W. (2018). The prognostic value of urinary chemokines at 6 months after pediatric kidney transplantation. *Pediatric Transplantation*.

[24] Malvezzi P., Fischman C., Rigault G. (2019). Switching renal transplant recipients to belatacept therapy: results of a real-life gradual conversion protocol. *Transplant Immunology*.

[25] Lo D. J., Weaver T. A., Kleiner D. E. (2011). Chemokines and their receptors in human renal allotransplantation. *Transplantation*.

[26] Li J., Koerner J., Basler M., Brunner T., Kirk C. J., Groettrup M. (2019). Immunoproteasome inhibition induces plasma cell apoptosis and preserves kidney allografts by activating the unfolded protein response and suppressing plasma cell survival factors. *Kidney International*.

[27] Yun J. J., Fischbein M. P., Whiting D. (2002). The role of MIG/CXCL9 in cardiac allograft vasculopathy. *The American Journal of Pathology.*.

[28] Rotondi M., Netti G. S., Lazzeri E. (2010). High pretransplant serum levels of CXCL9 are associated with increased risk of acute rejection and graft failure in kidney graft recipients. *Transplant International*.

[29] Jones B., Koch A. E., Ahmed S. (2012). Pathological role of fractalkine/CX3CL1 in rheumatic diseases: a unique chemokine with multiple functions. *Frontiers in Immunology*.

[30] Xu C. X., Shi B. Y., Jin Z. K. (2018). Multiple-biomarkers provide powerful prediction of early acute renal allograft rejection by combination of serum fractalkine, IFN-gamma and IP-10. *Transplant Immunology*.

[31] Gerszten R. E., Garcia-Zepeda E. A., Lim Y. C. (1999). MCP-1 and IL-8 trigger firm adhesion of monocytes to vascular endothelium under flow conditions. *Nature*.

[32] Weber C., Belge K. U., von Hundelshausen P. (2000). Differential chemokine receptor expression and function in human monocyte subpopulations. *Journal of Leukocyte Biology*.

[33] Hribova P., Lacha J., Kotsch K. (2007). Intrarenal cytokine and chemokine gene expression and kidney graft outcome. *Kidney & Blood Pressure Research*.

[34] Pengam S., Durand J., Usal C. (2019). SIRPalpha/CD47 axis controls the maintenance of transplant tolerance sustained by myeloid-derived suppressor cells. *American Journal of Transplantation*.

[35] Mueller F. B., Yang H., Lubetzky M. (2019). Landscape of innate immune system transcriptome and acute T cell-mediated rejection of human kidney allografts. *JCI Insight*.

[36] Mollica Poeta V., Massara M., Capucetti A., Bonecchi R. (2019). Chemokines and chemokine receptors: new targets for cancer immunotherapy. *Frontiers in Immunology*.

[37] Hsu W. T., Lin C. H., Jui H. Y. (2018). CXCR4 antagonist reduced the incidence of acute rejection and controlled cardiac allograft vasculopathy in a swine heart transplant model receiving a mycophenolate-based immunosuppressive regimen. *Transplantation*.

[38] Zou X. F., Gu J. H., Cui Z. L., Lu Y. W., Gu C. (2017). CXC chemokine receptor type 4 antagonism ameliorated allograft fibrosis in rat kidney transplant model. *Experimental and Clinical Transplantation*.

[39] Striz I. (2017). Cytokines of the IL-1 family: recognized targets in chronic inflammation underrated in organ transplantations. *Clinical Science*.

